# Design of a microstrip Wilkinson power divider using a low pass filter with the particle swarm optimization algorithm

**DOI:** 10.1038/s41598-024-66544-6

**Published:** 2024-07-31

**Authors:** Milad Mohammadi, Gholamreza Karimi, Hesam Ghitasy Sarabi

**Affiliations:** Electrical Department, Faculty of Electrical and Computer Engineering, Razi University, Kermanshah, 6714967346 Iran

**Keywords:** New resonators, Wilkinson power divider, PSO algorithm, Compact size, Isolation resistor, Engineering, Materials science, Physics

## Abstract

In this paper, a microstrip Wilkinson power divider (MWPD) based on particle swarm optimization (PSO) algorithm is designed, simulated, and fabricated using novel resonators. In addition, attenuators and open-ended stubs are incorporated to generate a broad cut-off band and reduce unwanted harmonics. The proposed power divider has a central frequency of 1 GHz. The performance of each used resonator is analyzed based on lumped-element circuit models.The *L* and *C* parameters of the equivalent circuit of the used resonators are predicted and optimized with the assistance of the PSO method. The subsequent phase was the fabrication of the proposed MWPD, after which its performance was evaluated in the light of the results obtained from the simulation. It was discovered that there was a high degree of concordance between the two. On the other hand, the fabricated circuit has several benefits, including a suitable S_12_ of − 3.15 dB, a high return loss of less than − 24 dB at the operating frequency, a compact size of 0.058 ${\varvec{\lambda}}_{{\varvec{g}}}$ × 0.064 ${\varvec{\lambda}}_{{\varvec{g}}}$, and the ability to remove undesired harmonics. The results show a high level of suppression of the unwanted harmonics (up to the 16th harmonic) and a great responsiveness in the passband, while having very low ripple. As a result, the proposed circuit may be used in a wide variety of electronic devices, such as radar transmitter and receiver circuits, and many other high-frequency systems.

## Introduction

The development of high-frequency and telecommunication circuits has played a crucial role in shaping modern society by revolutionizing communication, information exchange, and technological advancements. These circuits are at the heart of various devices and systems that enable the fast and efficient transmission of data over long distances^1–6^. Power dividers and combiners are now an part the integral elements of telecommunication systems and circuits, and their applications include wireless and Bluetooth communication systems, mobile phones, phase shifters, biomedical instruments, antenna arrays, etc. One of the main tasks of these passive circuits is to divide or combine the power of signals at radio frequencies^1^. There are several types of power dividers, such as Wilkinson^5^, Gysel^7,8^, and T-junction^9^, etc., each of which has advantages and disadvantages. However, among these structures, the Wilkinson structure is more suitable because of its compact size and the ability to provide a wider bandwidth^1^.

A combination of a ring coupler and a Gysel power divider is suggested in^10^ for an N-way planar power divider/combiner. The entire symmetrical divider is constructed on a two-layer printed circuit board in the given structure by substituting the common point of the standard Gysel power divider with a ring coupler. Despite its novel concept, this circuit’s fractional bandwidth (FBW) is only around 34.8%, and it fails to eliminate unwanted harmonics.

A small, three-port power divider is developed and fabricated in^11^. Harmonic suppression up to the ninth order is achieved using two-part resonators between the input and output ports and stepped impedance transmission lines. The power divider features a wide isolation band and a simple construction, according to this specific reference.

An MWPD that can dampen the 2nd, 3rd, and 4th harmonics has been suggested in^12^. The foundation of this MWPD is an asymmetric meta-structure. A combination of the spoofing surface plasmon polariton and defect ground structure is used to generate the meta-structure. Within the operating frequency range, it can suppress the second, third, and fourth harmonics in addition to realizing the impedance conversion function.

The article^13^ presents the study and fabrication of a high-performance MWPD with a low insertion loss and a large suppression factor at 7 GHz. To form the planned MWPD, two open stubs are replaced with regular transmission lines. Furthermore, even and odd mode analysis was used to investigate the characteristics of the recommended stub. However, it has a convoluted design and a low FBW, which is a major drawback.

Some of the presented research focuses on the geometrical structure of resonators. For example, rectangular resonators and U-shaped transmission lines are used in^14^. Another type of MWPD is presented in^15^ where trapezoidal-shaped resonators are used. The dimensions of the presented structure are very small, and transmission line analysis is used for the mathematical analysis of the presented circuit. In^16^, triangular-shaped resonators were used to generate a large stop band bandwidth. This structure has suppressed the undesirable 16 harmonics. However, its insertion loss level is not adequate, and it also has minimal isolation between the output ports.

While coupled-line and resonator-based power dividers work well to reduce size and decrease harmonics, but they also increase the insertion loss parameter. Moreover, open-stub-based power dividers have a simple construction and reasonable performance; nevertheless, this approach is not very good at reducing size or suppressing harmonics^17^.

On the other hand, several researchers have focused their attention on the mathematical study of the power divider. As part of the design process, the neural network model and the *LC*-equivalent circuit model are utilized to predict the transmission zeros of the power divider provided in^18^. The required harmonics can be suppressed using these transmission zeros. The suggested power divider is now more effective, and it is all down to the neural network model’s prediction of the major circuit parts. Also, in^19^, a surrogate neural network model was used to design a microstrip power divider and a low-pass filter (LPF). The suggested technique improves the performance and frequency response of a wide range of microwave devices. Their efficient design is a result of using the surrogate model of the proposed artificial neural network to determine the dimensions of the LPF and the power divider. However, the suggested structure has a restricted FBW and is difficult to produce due to its complexity. Ref.^20^ presents yet another approach that may be utilized to study and estimate the parameters of a power divider. In this study, the PSO method was utilized to compute the values of the equivalent inductors and capacitors in the microstrip structure, which resulted in an extremely optimum result. In addition, it has proved to be very accurate.

In the rapidly evolving landscape of microwave and radio frequency (RF) engineering, the design and optimization of MWPDs play a pivotal role in improving the performance of communication systems. The demand for efficient and compact MWPDs has led researchers to explore innovative designs and optimization techniques. This scientific article delves into the complex field of power divider design, presenting a novel approach that combines new resonators with the PSO algorithm to achieve superior performance characteristics. The center frequency of the proposed small-size structure is 1 GHz, and it suppresses 16 unwanted harmonics. Also, its FBW is 148% and 173% under the conditions of − 15 dB return loss and − 3 dB insertion loss. The design, simulation, fabrication, and measurement of the proposed circuit involves several key steps. Here’s a general outline of the process:

1. Problem formulation and specification: MWPD specifications and requirements definition. Determining the parameters of open stubs and new resonators in terms of their effect on the overall performance of the power divider
2. Initial circuit design: Designing the initial circuit based on the MWPD topology that incorporates new resonators and open stubs. Definition of the circuit elements, dimensions, and related materials for the resonators
3. PSO optimization: Implementation of the PSO optimization algorithm to fine-tune the LPF parameters.
4. Simulation: An electromagnetic simulation tool (ADS software) is used to simulate the performance of the designed MWPD. The validity of the optimized design is confirmed against the specified requirements and objectives.
5. Refinement and iteration: Based on the simulation results, the design is modified iteratively, adjusting the parameters and optimizing until the desired performance is reached.
6. PCB Layout and Fabrication: Translation of the optimized circuit design to the printed circuit board (PCB) design.
7. Measurement Setup: Setup a measurement environment with appropriate RF test equipment, including network analyzers and power meters.
8. Data Analysis and Comparison: Analyzing the measured data and comparing it with simulated results to validate the accuracy of the design.

## Conventional structure of the MWPD

**Figure 1:**
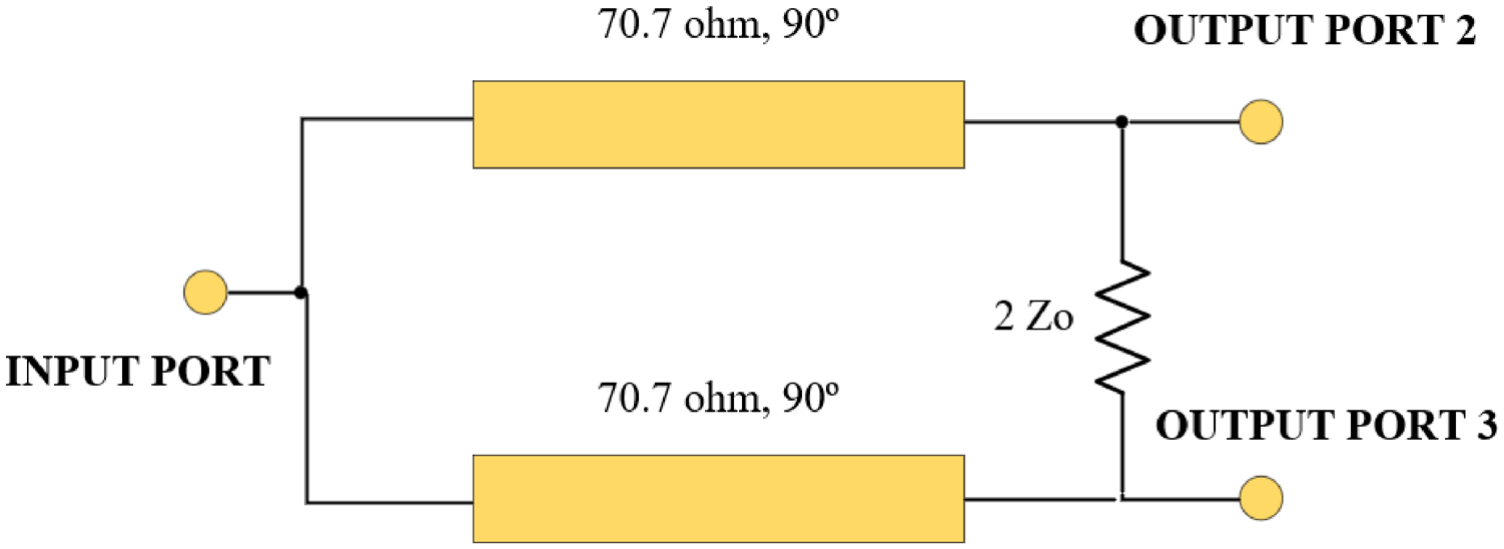
Wilkinson power divider’s functional block diagram^3^.

The design of the famous MWPD is shown in Fig. 1. It comprises two transmission-line sections with electrical lengths of 90º, characteristic impedances of 70.7 Ω, and one isolation resistor.

The MWPD can be implemented using microstrip or stripline technology and is typically used in applications where high isolation and low insertion loss are required, such as in satellite communication systems, radar systems, and wireless networks.

### Resonator design

In high-frequency circuits, resonators are used for various purposes such as filtering, tuning, and frequency stabilization. They can be used to select specific frequencies for transmission or reception, reject unwanted frequencies, or provide stable oscillations for signal generation. Resonators can be implemented using a number of different technologies, such as quartz crystals, ceramic materials, or *LC* (inductor-capacitor) circuits. Each technology has its advantages and limitations in terms of frequency range, stability, size, cost, and performance^1–3^.

**Figure 2:**
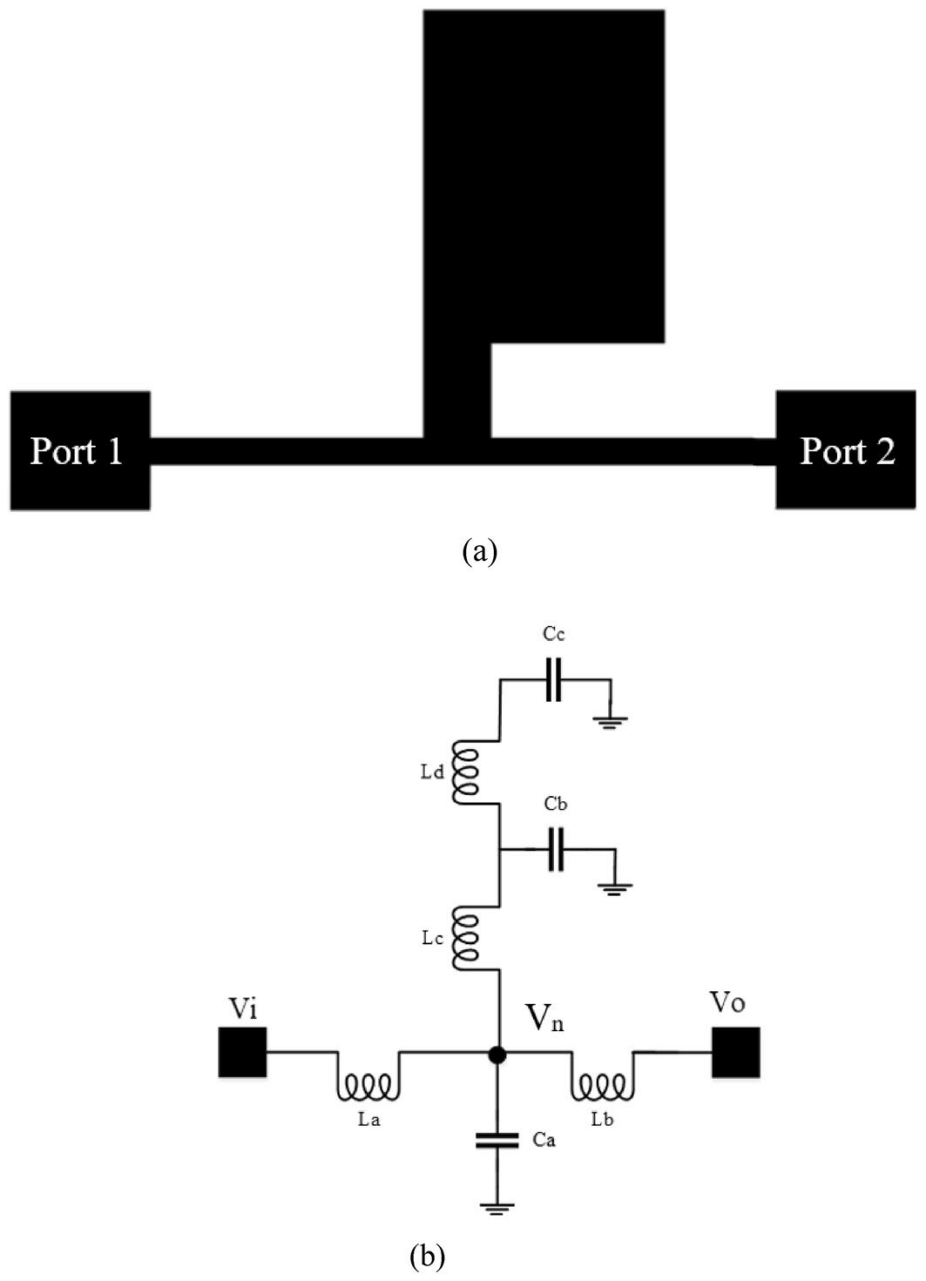
(**a**) Proposed microstrip resonator (**b**) *LC* equivalent circuit of the proposed resonator.

Here, we have used new microstrip resonators to improve the performance of the conventional MWPD. In Figs. 2a and b, the layout of the new resonator, and its *LC* equivalent circuit are shown, respectively.

Inductor and capacitor (*LC*) equivalent circuits may be designed for microstrip lines because of their shape. The performance of microstrip resonators can be easily analyzed with the help of this circuit, which produces a schematic diagram of the intended design^2^.

Therefore, the equivalent *LC* circuit of the proposed resonator is shown in Fig. 2b. Note that La is equal to Lb.

To apply the *LC* model to microstrip lines, consider each one as a ground capacitor and a series inductor. It is possible to extrapolate the matching *LC* circuit from here. Accurate reproductions of transmission lines can be made using the following formulae^2,12, 21^:

1 $$C = \left[ {8.85 \times 10^{ - 12} \left\{ {\left[ {\frac{{\varepsilon_{r} \times w}}{h}} \right]^{1.08} + \left[ {2\pi \left( {\frac{{\varepsilon_{r} + 1}}{2}} \right)\left( {\frac{1}{{\ln \left( {\frac{8h}{w} + 1} \right)}} - \frac{w}{8h}} \right)} \right]^{1.08} } \right\}^{0.926} } \right] \times l$$

2 $$L = \frac{Z \times l}{{V_{p} }} , V_{p} = \frac{c}{{\sqrt {\varepsilon_{re} } }}$$

For $$w/h \le 1$$:

3 $$\varepsilon_{re} = \frac{{\varepsilon_{r} + 1}}{2} + \frac{{\varepsilon_{r} - 1}}{2}\left\{ {\left[ {1 + 12\frac{h}{w}} \right]^{ - 0.5} + 0.04\left[ {1 - \frac{w}{h}} \right]^{2} } \right\} , Z = \frac{\eta }{{2\pi \sqrt {\varepsilon_{re} } }}{\text{ln}}\left[ {8\frac{h}{w} + 0.25 \frac{w}{h}} \right]$$

For $$w/h \ge 1$$:

4 $$\varepsilon_{re} = \frac{{\varepsilon_{r} + 1}}{2} + \frac{{\varepsilon_{r} - 1}}{2}\left\{ {\left[ {1 + 12\frac{h}{w}} \right]^{ - 0.5} } \right\} , Z = \frac{\eta }{{\sqrt {\varepsilon_{re} } }}\left\{ {\frac{h}{w} + 1.393 + 0.677\ln \left[ {\frac{w}{h} + 1.444} \right]} \right\}^{ - 1}$$

where Z is the characteristic impedance and Vp is the phase velocity of the transmission line. The constant speed of light, *c* is equal to (120π) 377Ω, *l* is the length of the transmission line, *w* is the width of the line, and *h* is the thickness of the substrate.

Based on Fig. 2b, we may assume that the observed impedance is from V_n_ is Z_n_ and derive the transfer function as follows:

5 $$\frac{V_{o}}{50} + \frac{{V_{o} - V_{n} }}{{S \times L_{b}}} = 0$$

6 $$V_{n} = \frac{{\left( {L_{b} \times S + 50} \right)V_{o} }}{50}$$

7 $$\frac{{V_{n} - V_{i} }}{{L_{a} \times S}} + \frac{{V_{n} }}{{Z_{n} }} + \frac{{V_{n} - V_{o} }}{{L_{b} \times S}} = 0$$

8 $$Z_{n} = (((( {\frac{1}{{C_{c} \times S}} + L_{d} \times S}) ||\frac{1}{{C_{b} \times S}}) + L_{c} \times S) ||\frac{1}{{C_{a} \times S}})$$

In this design, $L_{a} = L_{b}$. The transfer function between Vo and Vi is calculated using Eqs. (7) and (8) as follows:

9 $$\frac{{V_{o} }}{{V_{i} }} = \frac{{50 \times Z_{n} }}{{\left( {L_{a} \times S + 2Z_{n} } \right)\left( {50 + L_{a} \times S} \right) - \left( {50Z_{n} } \right)}}$$

**Figure 3:**
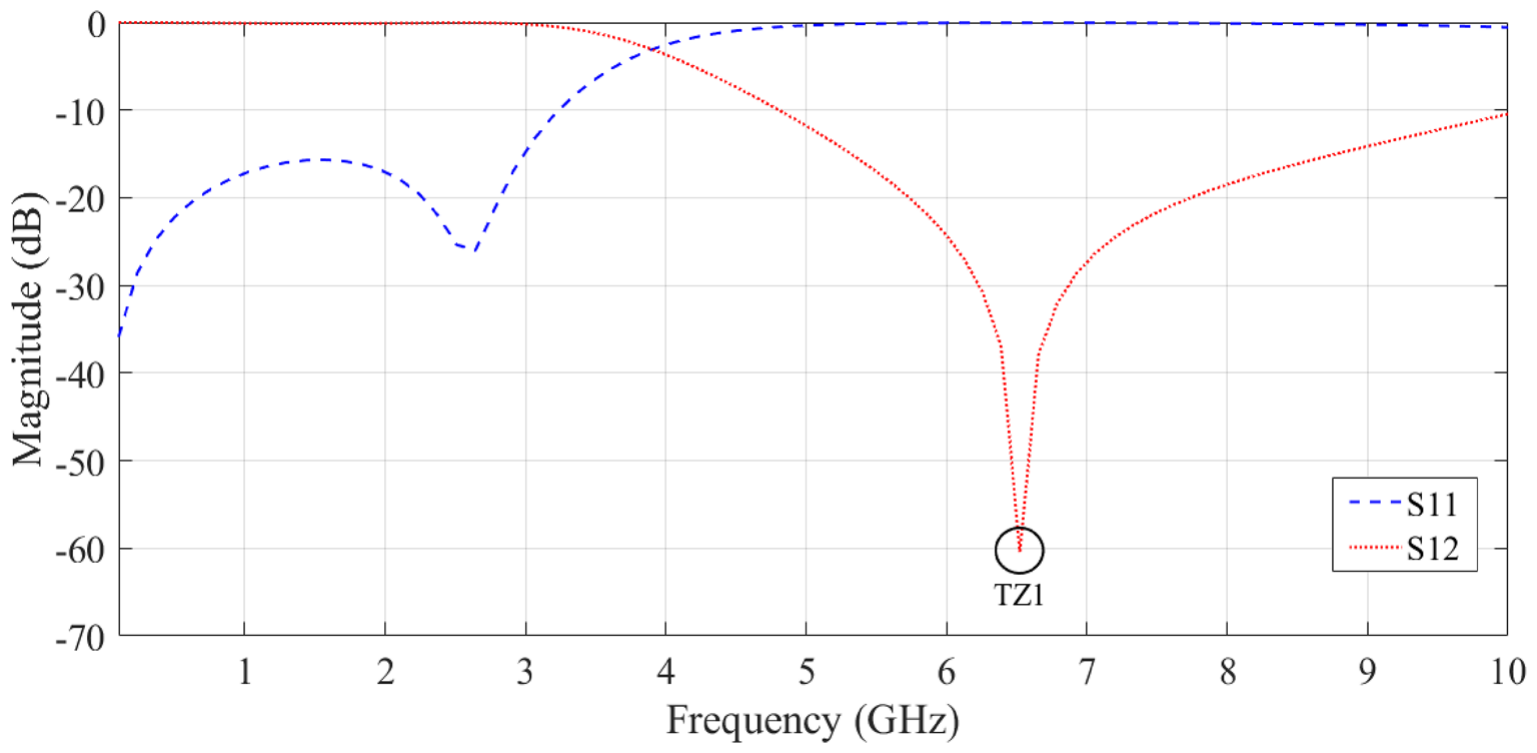
Simulation results of the of the S_12_ and S_11_ parameters of the proposed resonator.

The simulation results (EM analysis) of the proposed resonator in the structure of a basic Wilkinson power divider are shown in Fig. 3. At 6.6 GHz, the proposed resonator has a transmission zero. This resonator passes the input signal up to a frequency of about 3.2 GHz. Additionally, the S_11_ parameter value in the pass band is less than − 10 dB.

The result in Fig. 3 shows that the resonators used create a transmission zero in the frequency response of the proposed power divider. However, we need to use resonators and other suppressors to improve the frequency response of the circuit to increase the bandwidth and eliminate the unwanted harmonics. Note that the proposed final circuit is placed on the main transmission line of the primary Wilkinson power divider. Indeed, we replace the primary transmission line of the circuit with a filter structure.

### New suppressor

**Figure 4:**
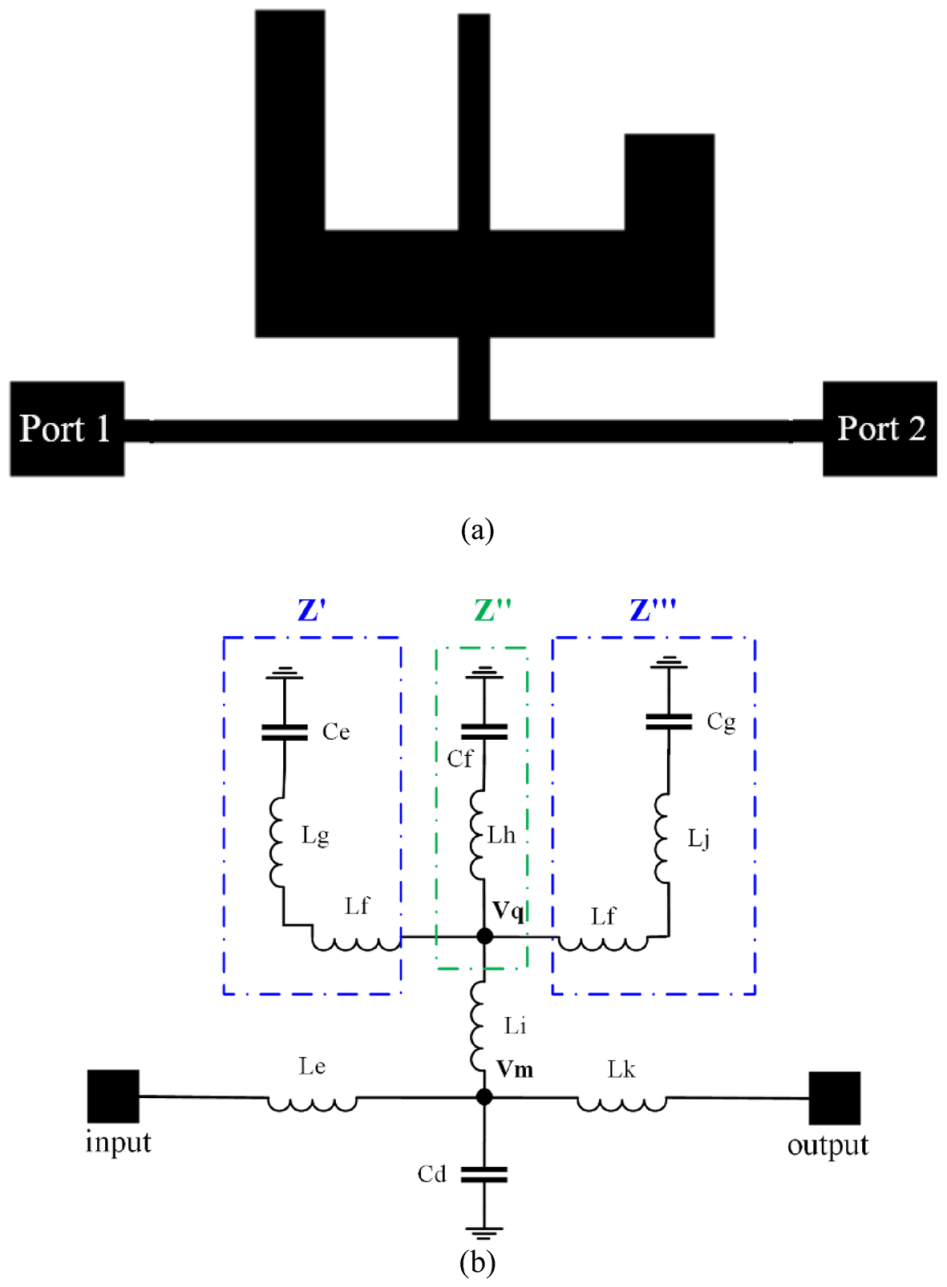
(**a**) Proposed suppressor (**b**) *LC* equivalent circuit of the proposed suppressor.

We modified the design of the proposed circuit, as shown in Fig. 4, to increase the stop band bandwidth and obtain a broad stop band. To provide a good stop band frequency response for the LPF, a new suppressor has been employed. Figure 4 depicts how several TZs are formed in the stop band as a result of the designed suppressor.

The LPF design is achieved by connecting the designed circuit to a suppressor. This suppressor is used to reduce the stop band S_12_ parameter. The S_12_ parameter should be reduced in the stop band of an LPF so that no signal passes through it. Our approach provides an innovative and straightforward solution to extend the stopping range. The *LC* equivalent circuit of the proposed suppressor is shown in Fig. 4b.

In Fig. 4b, *Le* to *Lk* and *Cd* to *Cg* are the inductances and capacitances of the transmission lines, respectively. In this design, the equivalent impedances at nodes Vq, and Vm are Zq, and Zm, respectively. So, using KCL and KVL relations between circuit elements, it can be written as follows:

10 $$\frac{V_{o}}{50} + \frac{{V_{o} - V_{m} }}{{S \times L_{k}}} = 0$$

11 $$V_{m} = \frac{{\left( {L_{e} \times S + 50} \right)V_{in} }}{50}$$

12 $$\frac{{V_{m} - V_{in} }}{{L_{e} \times S}} + \frac{{V_{m} }}{{Z_{m} }} + \frac{{V_{m} - V_{out} }}{{L_{k} \times S}} = 0$$

13 $$Z_{m} = (( {\frac{1}{{C_{d} \times S}}} )||\left( {L_{i} \times S + Z_{q} } \right))$$

$$\frac{1}{{Z_{m} }} = \frac{1}{{\frac{1}{{C_{d} \times S}}}} + \frac{1}{{L_{i} \times S + Z_{q} }}$$

14 $$Z^{\prime} = \left( {\frac{1}{{C_{e} \times S}}} \right) + L_{g} \times S + L_{f} \times S$$

15 $$Z^{\prime\prime} = \left( {\frac{1}{{C_{f} \times S}}} \right) + L_{h} \times S$$

16 $$Z^{\prime\prime\prime} = \left( {\frac{1}{{C_{g} \times S}}} \right) + L_{j} \times S + L_{f} \times S$$

17 $$\frac{1}{{Z_{q} }} = \frac{1}{{Z^{\prime\prime\prime}}} + \frac{1}{{Z^{\prime\prime}}} + \frac{1}{{Z^{\prime}}}$$

18 $$Z_{q} = \frac{\frac{1}S}{{\frac{c_e}{1+(L_g+L_f)c_eS^2}}+{\frac{c_f}{1+L_hc_fS^2}}+{\frac{c_g}{1+(L_j+L_f)c_gS^2}}}$$

In this circuit $L_{e} = L_{k}$. The transfer function between Vo and Vi can be calculated using Eqs. (10) to (18) as follows:

19 $$\frac{{V_{out} }}{{V_{in} }} = \frac{{\left( {L_{e} \times S + 50} \right) \times \left( {2 \times Z_q + L_{e} \times S} \right)}}{{50 \times Z_q }} - 1$$

**Figure 5:**
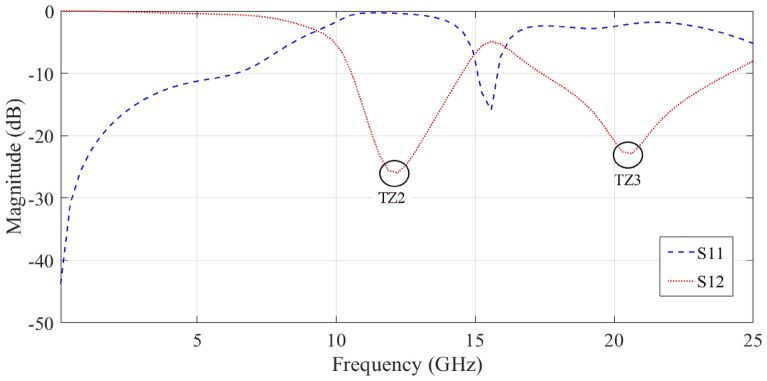
Result of the simulation of the S_12_ and S_11_ parameters of the proposed suppressor.

The simulation result (EM analysis) of the proposed suppressor in the structure of a basic Wilkinson power divider is shown in Fig. 5. As expected, it produced two transmission zeros at around 11.4 and 20.8 GHz.

The layout module of the Advanced Design System (ADS) software models the resonator and suppressor shown in Figs. 2a and 4a using the Rogers 5880 substrate (h = 20 mil, tan d = 0.0009, and ε_r_ = 2.2). Consequently, for each section of the microstrip line, we know the dimensions of the resonator and suppressor, namely their length (l) and width (w). In contrast, the value of the inductor and capacitor corresponding to this line may be easily determined using Eqs. (1) to (4). This is done for each transmission line based on the equivalent circuit shown in Figs. 2b and 4b. This is because all the important criteria are available. Equations (1) to (4) are used to determine the initial values of these capacitors and inductors in Table 1.

**Table 1:** Initial values of equivalent inductors and capacitors (unit: L: nH; C: pF).

*La*	*Lb*	*Lc*	*Ld*	*Lf*	*Lg*	*Lh*	*Li*	*Lj*	*Lk*
0.045	7.8	3.2	4.08	2.23	1.27	0.55	1.84	0.45	2.08

*Ca*	*Cb*	*Cc*	*Cd*	*Cf*	*Cg*				
0.055	0.25	0.48	0.023	1.34	1.01				

Figures 3 and 5 demonstrate that the return losses of the proposed circuit are unsuitable in both the passband and the cut-off band, and that their cut-off bandwidth is limited. Therefore, mathematical optimization algorithms such as PSO can be used to calculate the most optimal result.

## Estimation of parameters and optimization

PSO has been used to determine optimal design parameters because it performs well in designing microwave circuits^22^. Equation (20) shows the best design objective function.

20 $$F = {\text{min}}[\mathop \sum \limits_{i = 1}^{2} \frac{{S_{11} .f\left( i \right)}}{n}\left] { + {\text{max}}} \right[\mathop \sum \limits_{i = 1}^{2} \frac{{S_{12} .f\left( i \right)}}{n}]$$

To design an ultra-wide bandwidth power divider, we need to use a two-part Eq. (20). At the operating frequencies of the power divider, the insertion loss should reach its maximum value (-3 dB) in the objective function while minimizing the input/output return loss. As a result, the optimal parameters of the power divider are those found by applying these requirements. Optimization techniques are the best choice to determine them.

PSO is an advanced algorithm in the field of cumulative intelligence, introduced in^23^. In recent years, the PSO algorithm has received much attention due to its simplicity and ease of use^24–26^.

The PSO algorithm is a computational algorithm developed based on the group behavior of insects and birds in search of food. This algorithm is used to solve complex and non-linear optimization problems. Particles in the PSO algorithm move around their current location in the search space at their own speed. They then update their speed and location based on their performance and the performance of the best particle so far, as well as the best location they have found so far. This process continues iteratively until they reach the optimal point in the search space.

By using the PSO algorithm, complex and non-linear optimization problems can be solved.

The search process requires two memories for each particle, one of which is used to maintain the optimal position of the particle. The particles decide on their next steps based on the data they extract from these memories. In each iteration, the velocity and position of each particle are updated based on the best absolute and local solutions available^27^. By adding the velocity of the particle to its current position, the location of the population may be determined.

21 $$X_{j} \left( i \right) = X_{j} \left( {i - 1} \right) + V_{j} \left( i \right)$$

$X_{j} \left( i \right)$ indicates the position of particle j, $V_{j} \left( i \right)$ indicates the velocity and i indicates the number of times the velocity variable is repeated. The velocity can be determined using the following equation:

22 $$V_{j} \left( i \right) = \theta \left( i \right) \times V_{j} \left( {i - 1} \right) + c_{1} r_{1} \left[ {P_{best,j} - X_{j} \left( {i - 1} \right)} \right] + c_{2} r_{2} \left[ {G_{best} - X_{j} \left( {i - 1} \right)} \right]$$

$V_{j} \left( i \right)$ is the i-th component of the velocity of particle j, r_1_, and r_2_ are uniform random values distributed in the interval (0,1), and based on experiments^27^, the parameters c_1_ and c_2_ represent individual and group learning components.

The particle’s local best position, $P_{best,j}$, and global best position, $G_{best}$ are also given. $\theta \left( i \right)$ is the inertial weight used to adjust the particle velocity in controlled laboratory experiments. From^28^, we can derive $\theta \left( i \right)$:

23 $$\theta \left( i \right) = \theta_{max} - \left( {\frac{{\theta_{max} - \theta_{min} }}{{i_{max} }}} \right)i$$

$\theta_{min} { }$ is the minimum number of iterations in the algorithm and $\theta_{max}$ is the maximum number of iterations. $\theta_{min}$ and $\theta_{max}$ are the minimum and maximum inertia weights, respectively. Experimental results often show that $\theta_{min}$ = 0.4 and $\theta_{max}$ = 0.7 are optimal^29^.

The design parameters for this work were optimized using the PSO technique with the values given in Table 2.

**Table 2:** Adjusted parameters of the PSO algorithm for the proposed power divider.

Inputs	Value
N	75
$\theta_{\max } ,\theta_{\min }$	0.7,0.4
$i_{\max }$	350
$c_{1} ,c_{2}$	2,2

**Figure 6:**
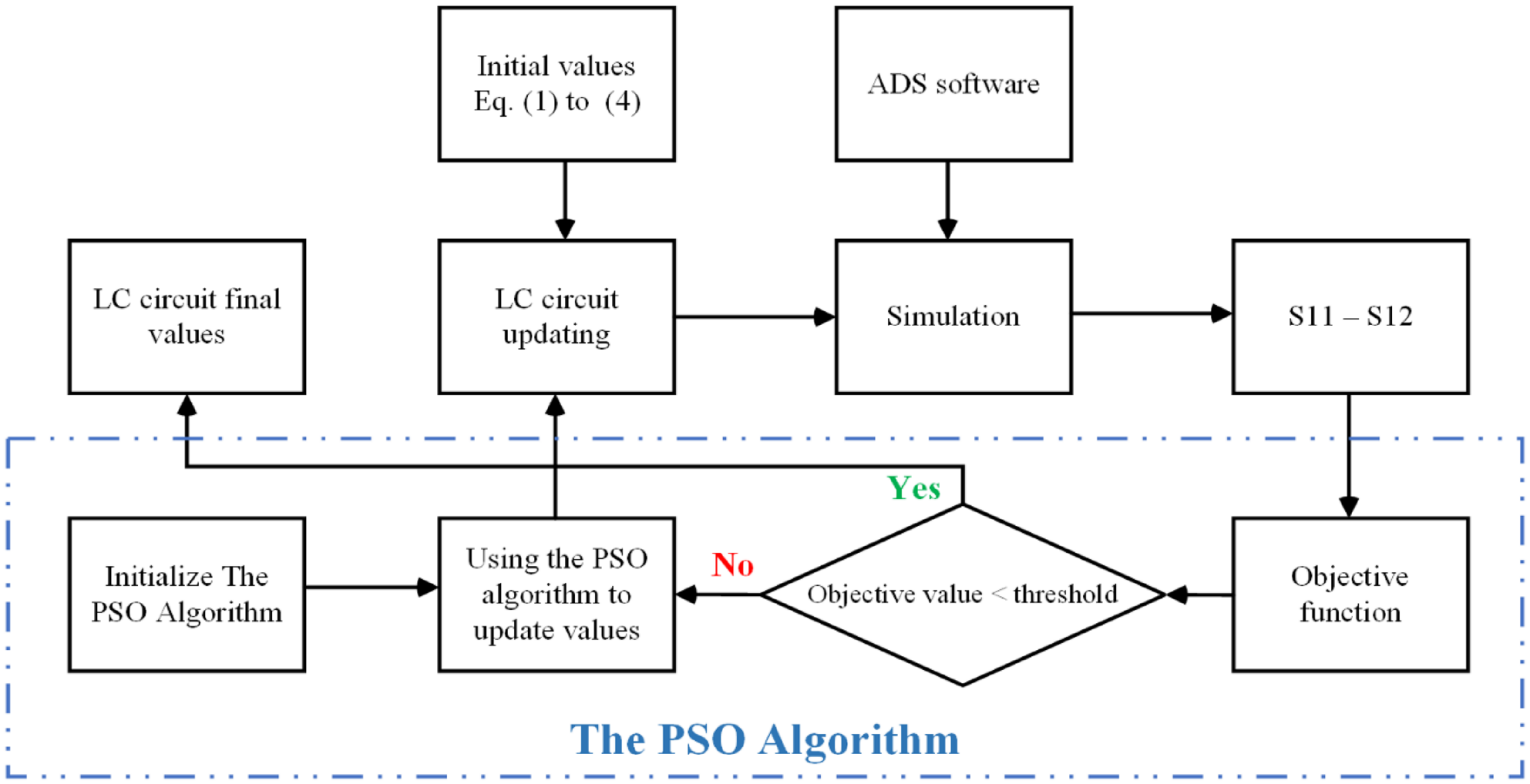
PSO algorithm process.

Once the overall design of the proposed resonator and suppressor has been finalized, the next step is to improve the circuit’s performance by finding the optimal values for the inductors and capacitors. The optimization of the structure is achieved by the utilization of an intelligent technique using the co-simulation of MATAB-ADS in conjunction with algorithms. This is done due to the complexity and inaccuracy of the conventional calculating approach for the circuit. The gain of these circuits is determined by the calculations represented by Eqs. (8) to (19). Figure 6 shows the implementation process of the PSO algorithm and the MATAB-ADS co-simulation approach. The optimization steps in this approach are as follows:

1. As a first step, set the initial values for the capacitors and equivalent inductors on the microstrip lines. Reduce the bird pack’s search area by defining a more precise optimal range for these characteristics. These initial values are obtained using Eqs. (1) to (4).
2. To obtain the S-parameters data, a simulation is started by calling ADS. The subsequent procedure involves extracting the pertinent data from the export and subsequently importing them back into MATLAB. The value of the objective function may be determined based on the variables S_12_ and S_11_. The objective function is expressed by Eq. (20).
3. A threshold is checked by the particle swarm optimizer to see if the objective function is satisfactory. In this instance, the PSO algorithm should be implemented to update 75 sets of *LC* parameters, which cover the entire location of the group.
4. Use ADS to obtain the S-parameters for the 75 groups of *LC* parameters that have been modified. Find the best outcomes from the 75 groups, then return to step three.
5. Keep exporting the optimized inductor and capacitor values until the specified threshold is reached.

In PSO algorithm loops, the parameters of the circuit are changed repeatedly until the performance of the circuit is adjusted to meet the specified objectives. Table 3 displays the optimal values of the circuit’s inductors and capacitors.

**Table 3:** Values for *L* and *C* obtained from the power divider provided based on the PSO method (unit: L: nH; C: pF).

*La*	*Lb*	*Lc*	*Ld*	*Lf*	*Lg*	*Lh*	*Li*	*Lj*	*Lk*
0.041	7.1	2.1	3.77	1.2	1.14	3.5	1.98	2.41	2.54

*Ca*	*Cb*	*Cc*	*Cd*	*Cf*	*Cg*				
0.038	0.094	1.02	0.007	0.021	0.017				

According to the values in Table 3, Eqs. (9) and (18) can be obtained.

24 $$T_{z1} = 6.62{ }GHz$$

25 $$T_{z2} = 11.36 GHz$$

26 $$T_{z3} = 20.75 GHz$$

The transmission zero (T_Z1_) for the proposed circuit is shown in Fig. 3 to be approximately 6.6 GHz, whereas the T_Z1_ for the arrangement is approximately 6.62 GHz. The findings of the layout and the presented circuit are in good agreement. Also, the calculated T_Z2_ and T_Z3_ were almost equal to the simulation results. In other words, placing the proposed resonator and suppressor together will produce a semi-LPF function.

**Figure 7:**
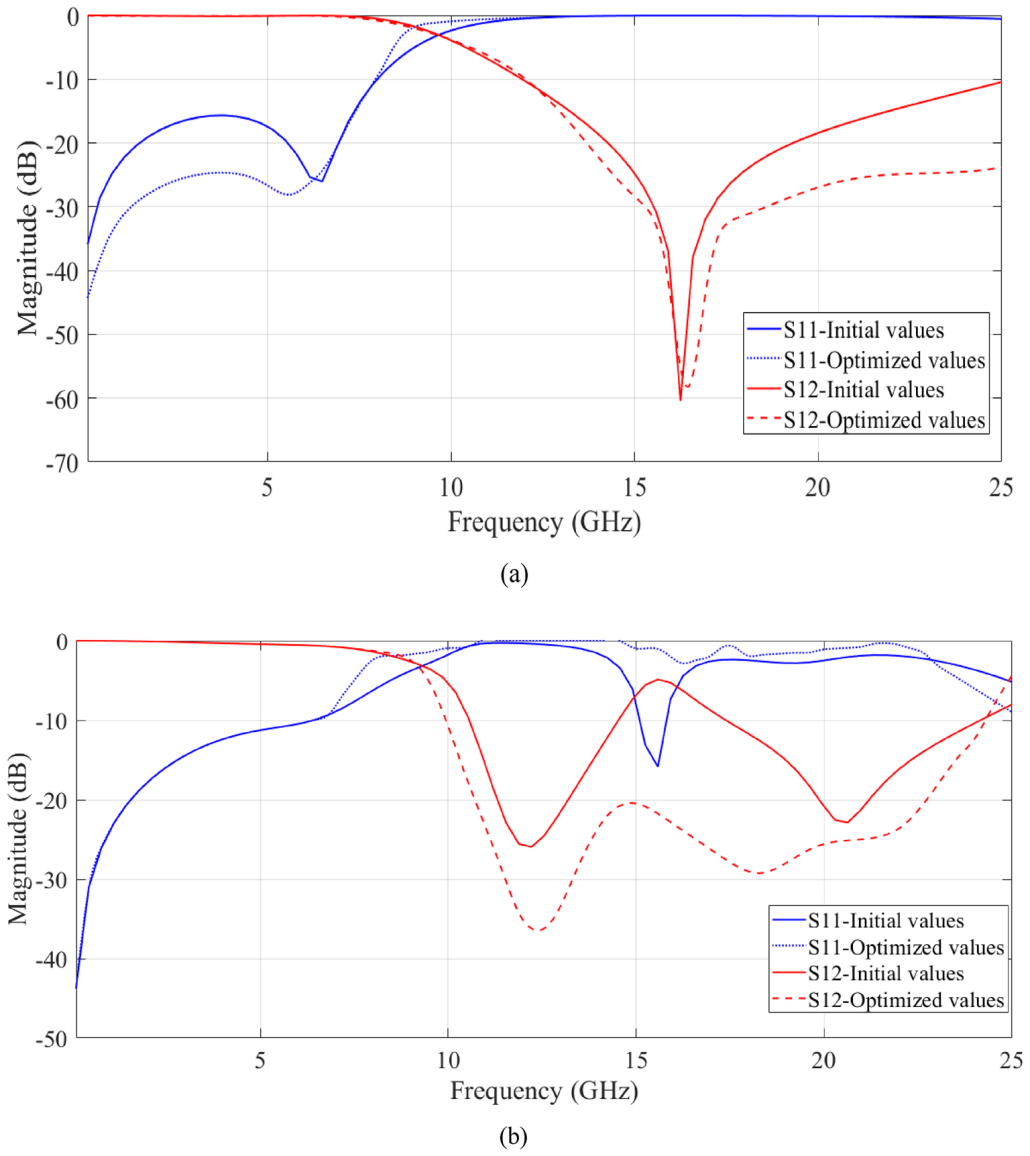
Simulation results of the scattering parameters with initial and optimized values for (**a**) designed resonator, and (**b**) designed suppressor.

Figures 7a and b respectively show the simulation results of the S-parameters of the resonator and suppressor circuits with optimized values. These results have been compared with the simulation results obtained using the initial values. As it is known, when the circuit elements have optimized values, a more suitable passband and cut-off band have been created. According to Fig. 7a, in the optimized mode, the S_12_ response is sharper and has created a wide cut-off band up to − 25 GHz with a level below − 24 dB. Also, the S_11_ level is lower in the pass band (− 22 dB). The results in Fig. 7b also show that the suppressor circuit with the best values from frequency 11.2 to 23.3 GHz has an S_12_ value of less than − 20 dB, which is much better than the circuit with the initial values. The S_11_ parameter in the cut-off band has a value close to zero. Thanks to the optimisation process, the developed circuit really has a superior response.

## Final structure of the designed MWPD

In order to achieve ultra-wide bandwidth and a suitable operating frequency, new resonators have been used symmetrically and inverted on both sides of the main transmission line of the circuit. A suppressor is also used to weaken unwanted signals. In the present design, the coupling effects of nearby lines are ineffective and are ignored in the calculations. On the other hand, the small stubs at the end have been used to create a wide cutting band along the transmission line.

**Figure 8:**
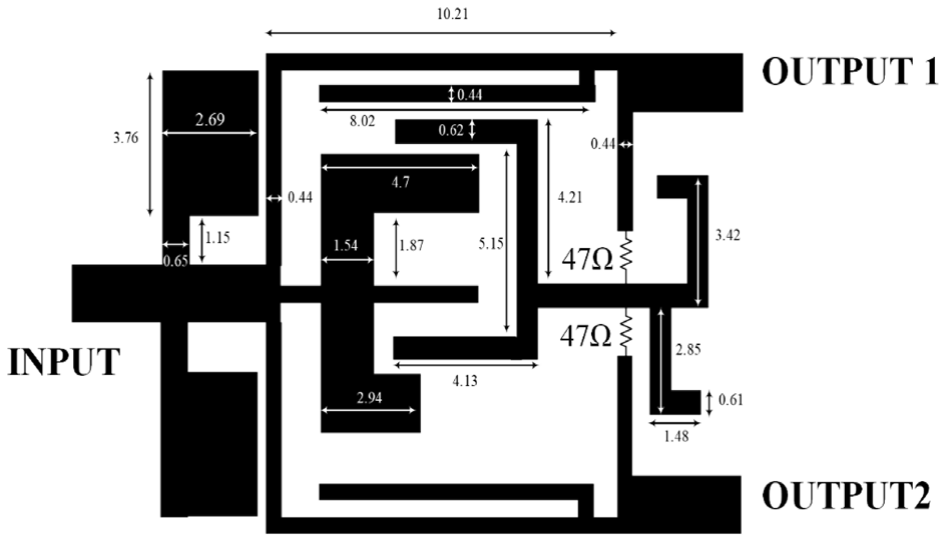
Final design of the proposed MWPD.

The final design of the MWPD is shown in Fig. 8. The important point and one of the innovations used in this layout is the use of two 47-Ω SMD resistors instead of the conventional 100-Ω isolation resistor in the MWPD structure. Also, the dimensions of the resonators, suppressors, and open-ended stubs in millimeters are shown in Fig. 8.

Due to the positioning of the stubs between the output ports, it is not feasible to insert a resistor in the redesigned arrangement depicted in Fig. 8. Consequently, it is necessary to employ two resistors in order to separate the output signals from one another.

**Figure 9:**
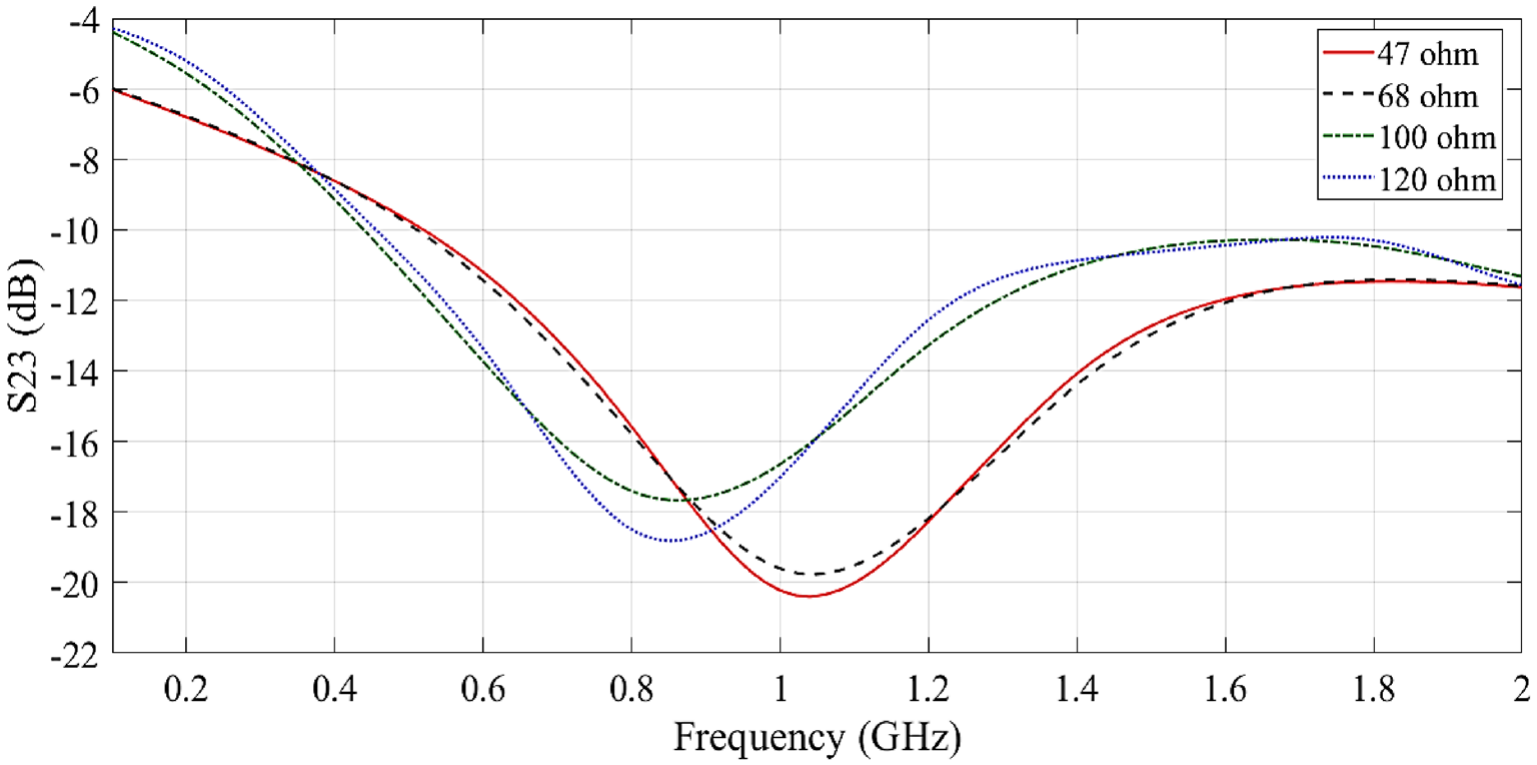
Simulation results of S_23_ for different values of isolation resistance.

Figure 9 displays the simulation results of S_23_ for various nominal levels of isolation resistance. From the simulation results, it can be observed that the highest level of isolation between the two output ports is achieved when the resistance is set to 47-Ω at the center frequency.

### Fabrication of the proposed MWPD

**Figure 10:**
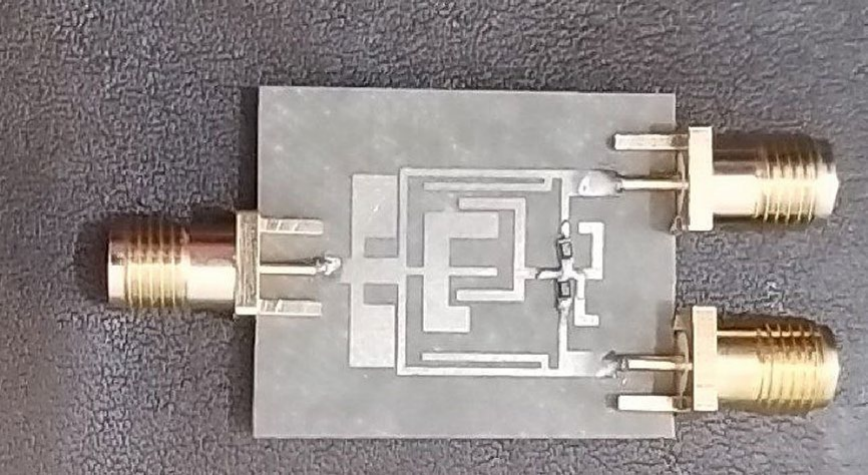
Fabricated MWPD.

After designing and simulating the proposed MWPD and calculating its parameters, the designed power divider is fabricated. This designed circuit is fabricated on a Rogers 5880 substrate with a thickness of 20 mil, a loss of 0.0009, and ε_r_ = 2.2. Figure 10 shows the final circuit. In this structure, two 47-Ω resistors are used, following the conventional structure of the MWPD, which improves the thermal conductivity of the circuit. It should be noted that ADS software was used for the design and simulation, and the fabricated power divider was measured by the B8510 network analyzer. Also, the fabricated circuit is shown in Fig. 10.

### Results and discussion

**Figure 11:**
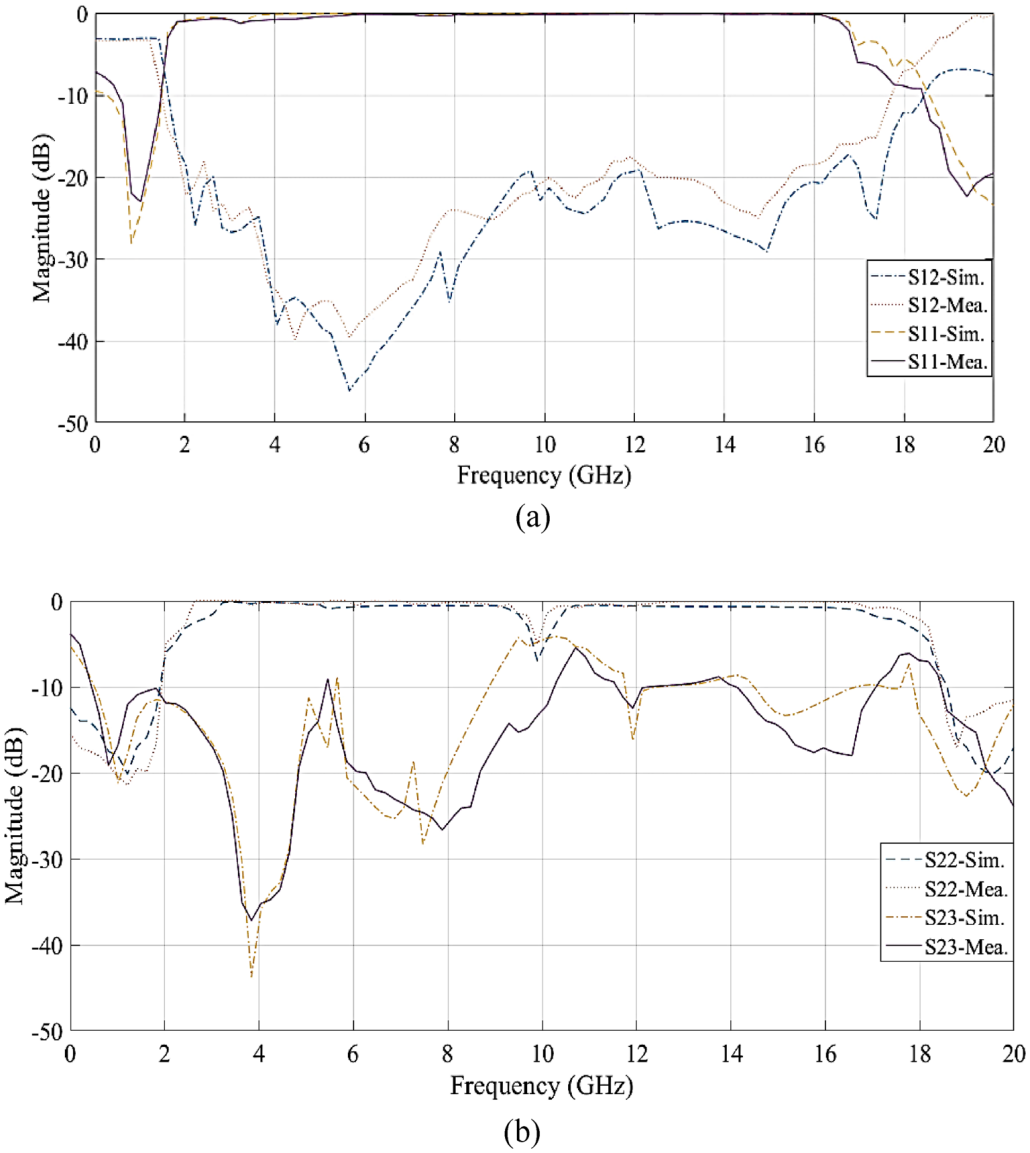
Simulation and measurement results (**a**) S_11_, S_12_, and (**b**)S_22_, S_23_.

This novel power divider features a small size (12.9 mm × 14.2 mm, or 0.058 ${\varvec{\lambda}}_{{\varvec{g}}}$ × 0.064 ${\varvec{\lambda}}_{{\varvec{g}}}$), excellent bandwidth, ideal return loss, and an operating frequency of 1 GHz. Also, the results of the simulation and measurement of parameters S_11_ and S_12_ are shown in Fig. 11a. Figure 11a shows that in the range of 0.32 GHz to 1.8 GHz, the proposed structure has S_11_ with an attenuation level below − 15 dB. Therefore, the FBW in the range where the return loss is less than − 15 dB is equal to 148% and in the range where the insertion loss is − 3 dB is equal to 173%, which is very ideal. Also, the unwanted harmonics of this circuit have been removed, up to 16 unwanted harmonics with a level below − 19.5 dB. The value of S_12_ in the second to eighteenth harmonics is equal to: − 20.5, − 26.6, − 37.8, − 38.4, − 44.1, − 36.5, − 32.1, − 23.6, − 21.3, − 23.3, − 19.5, − 27.4, − 28.1, − 24.9, and − 20.5 (all in dB), respectively.

The presented results show that the operating band is extremely wide. At the operating frequency (1 GHz), the value of S_12_ is approximately − 3.15 dB, which is very suitable, and the passband of the power divider is relatively flat and does not fluctuate much.

Figure 11b shows the results of the simulation and measurement of the output return loss (S_22_) and isolation between the parameters of two output ports (S_23_). The obtained measurements show that the value of S_22_ is approximately − 21 dB and that of S_23_ is approximately − 19 dB at the operating frequency of the designed MWPD. As a result, the output port of the power divider exhibits zero return loss, and the operational performance of the power divider ensures complete isolation between the two output ports.

Figures 11a and b show that the simulation and measurement results are very close to each other with only small differences. Possible reasons for these differences include connection losses, substrate characteristics, and the soldering of isolation resistors. Therefore, the fabricated circuit is quite practical and effective.

**Figure 12:**
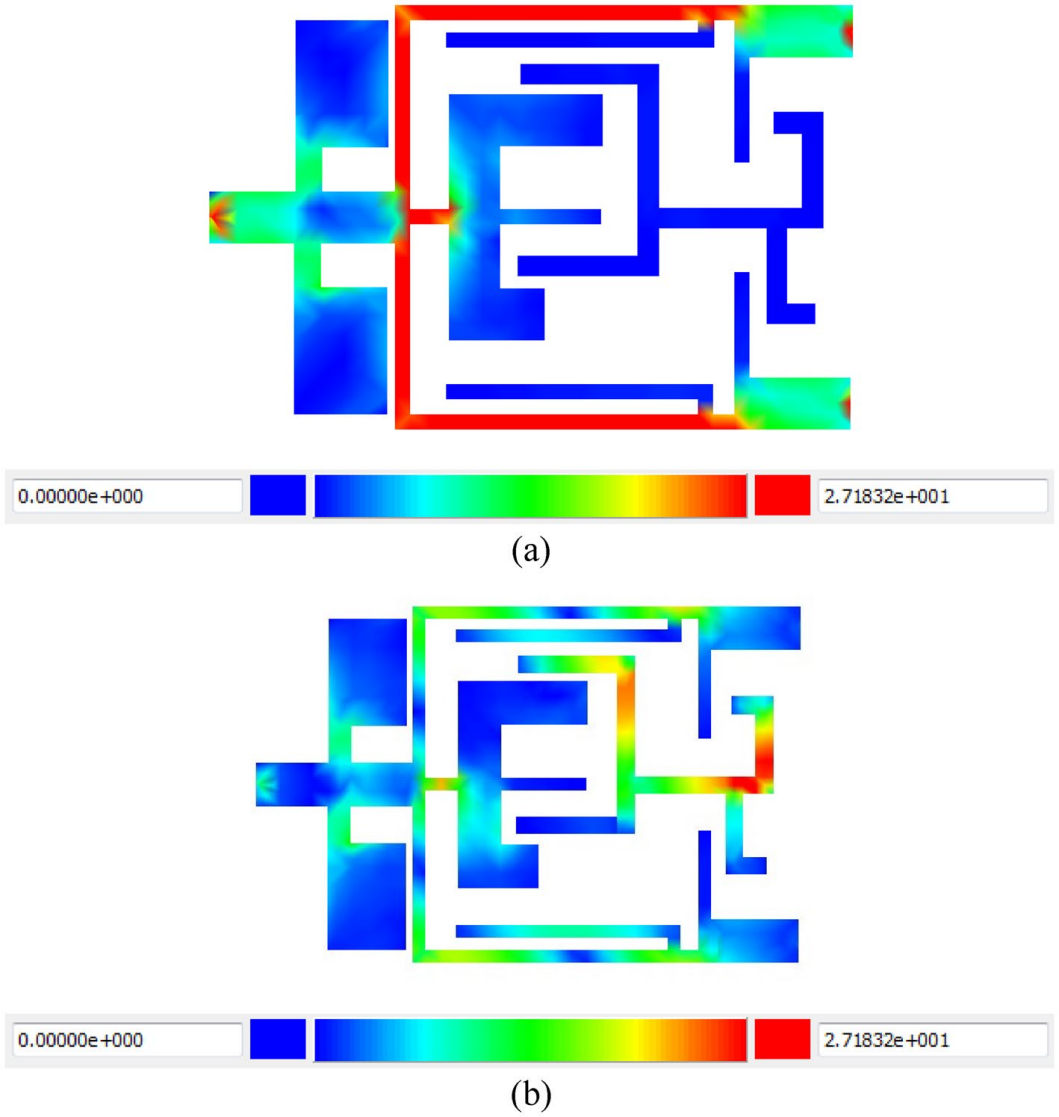
Current density distribution at (**a**) 1 GHz and (**b**) 6 GHz frequencies.

The current density distribution is depicted in Figs. 12a and b, respectively, for frequencies of 1 and 6 GHz. At a frequency of 1 GHz, as is generally accepted, we have an appropriate distribution of the electromagnetic field in the output ports, and the signal is allowed to pass. However, at a frequency of 6 GHz, which is in the cut-off band of the filter, the designed resonators and stubs prevent the signal from passing, and nothing at all is allowed to reach the output.

The final step was to compare the MWPD obtained using the filter circuit and the PSO optimization algorithm with other similar works that have been published. The results of this comparison are presented in Table 4.

**Table 4:** Comparison of the designed power divider with other works.

Refs.	Working frequency (GHz)	FBW (%)	S_11_ (dB)	S_12_ (dB)	Size (${\varvec{\lambda}}_{{\varvec{g}}}$ × ${\varvec{\lambda}}_{{\varvec{g}}}$)	Harmonic suppressed
^15^	0.58	–	− 17	− 3.2	0.2 × 0.109	2nd to 10th
						− 16 dB
^16^	1.65	(RL-15 dB) 68	− 20	− 3.21	0.066 × 0.069	2nd to 8th
						− 15 dB
^30^	2.45	(IL-3 dB) 19.6	− 19.5	− 3.59	0.25 × 0.38	2nd to 3rd
						− 19 dB
^31^	2.85	(RL-15 dB) 52.9	− 20	− 3.62	0.41 × 0.47	2nd to 4th
						− 20 dB
^32^	2.45	(RL-20 dB) 28	− 15	− 3.3	0.44 × 0.62	–
^33^	1.75	(RL-20 dB) 62.3	− 20	− 3.62	0.3 × 0.4	2nd to 4th
						− 20 dB
^34^	1	(RL-15 dB) 58	− 25.7	− 3.8	0.57 × 0.3	–
^35^	1	(IL-3 dB) 129	− 27.9	− 3.2	0.19 × 0.25	2nd to 3rd
						− 15 dB
^36^	5.99	(RL-20 dB) 13.5	− 20	− 4.05	0.77 × 0.77	2nd to 3rd
		(IL-3 dB) 25				− 20 dB
^37^	1.5	(RL-15 dB) 28	− 20	− 3.2	0.15 × 0.05	3ed to 6th
						− 20 dB
**This work**	**1**	**(RL-15 dB) 148**	− **24**	− **3.15**	0.058 × 0.064	**2nd to 16**^**th**^
		**(IL-3 dB) 173**				− **19.5 dB**

As can be seen, the circuit presented here is better in terms of FBW than all those mentioned in Table 4 and has the highest value. The S_12_ parameter has the most ideal conditions compared to the rest of the articles, and the dimensions of the designed circuit are relatively small and suitable considering that the circuit arrangement is in the Wilkinson form. In addition, this circuit has removed up to 16 disturbing harmonics. The designed MWPD shows great promise for various applications within the realm of microwave engineering, including radar systems, phased array antennas, aerospace, and defense.

The main novelty of this work is the use of two isolation resistors and a new suppressor. Furthermore, for the first time, the PSO algorithm has been used to calculate the *LC* equivalent circuit parameters of an MWPD to achieve improved performance in terms of insertion loss, input return loss, and isolation compared to traditional designs.

## Conclusion

In this paper, a compact 1 GHz MWPD with new resonators and suppressors was proposed. The incorporation of these resonators has introduced a fresh perspective on geometric configurations, resulting in tangible improvements in key performance metrics. To obtain the parameters of the presented circuit, the PSO method was used. Also, in this article, two 47-Ω isolation resistors are used between the output ports. This innovation increased FBW by 148% and 173%, improved S_12_ by − 3.15 dB, and reduced S_11_ by − 24 dB to address critical challenges in traditional power divider designs. The dimensions of this circuit are only 0.058 ${\varvec{\lambda}}_{{\varvec{g}}}$ × 0.064 ${\varvec{\lambda}}_{{\varvec{g}}}$. In addition, the presented structure suppresses the unwanted 16th harmonic, so it can be used in many RF and telecommunication systems.

## Data Availability

The datasets generated during the study are available from the corresponding author on reasonable request.
